# The micromass formation potential of human adipose-derived stromal cells isolated from different various origins

**DOI:** 10.1186/s13005-018-0178-0

**Published:** 2018-09-26

**Authors:** Benedikt Kleineidam, Sonja Sielker, Marcel Hanisch, Johannes Kleinheinz, Susanne Jung

**Affiliations:** 1 Department of Oral Surgery, University Hospital Bonn, Bonn, Germany; 2 Department of Cranio-Maxillofacial Surgery, Research Unit Vascular Biology of Oral Structures (VABOS), University Hospital Muenster, Waldeyerstraße 30D-48149, Muenster, Germany

**Keywords:** HADSC, Multipotency, Micromass, Regeneration

## Abstract

**Background:**

Adult stem cells appear to be a promising subject for tissue engineering, representing an individual material for regeneration of aged and damaged cells. Especially adipose derived stromal cells (ADSC), which are easily to achieve, allow an encouraging perspective due to their capability of differentiating into miscellaneous cell types. Here we describe the in vitro formation of human subcutaneous, visceral and omental ADSC micromasses and compare their histological attributes while being cultivated on collagen membranes.

**Methods:**

Subcutaneous, visceral and omental fat tissue derived cells were isolated and processed according to standard protocols. Positively stained cells for CD13, CD44 and CD90 were cultivated on agarose in order to study micromass formation using a special method of cell tracking. Stained paraffin-embedded micromasses were analysed morphologically before and after being plated on collagen membranes.

**Results:**

The micromass formation process was similar in all three tissue types. Subcutaneous fat tissue derived micromasses turned out to develop a more homogeneous and compact shape than visceral and omental tissue. Nevertheless all micromasses adhered to collagen membranes with visible spreading of cells. The immune histochemical (IHC) staining of subcutaneous, visceral and omental ADSC micromasses shows a constant expression of CD13 and a decrease of CD44 and CD 90 expression within 28 days. After that period, omental fat cells don’t show any expression of CD44.

**Conclusion:**

In conclusion micromass formation and cultivation of all analysed fat tissues can be achieved, subcutaneous cells appearing to be the best material for regenerative concepts.

## Background

Adipose derived stromal cells (ADSC) are multipotent cells well known in the literature. They occur in abundance, are easily to achieve and able to differentiate into miscellaneous lineages like osteoblasts, endothelial cells, or neurogenic cells without difficulties. [1] These special characteristics contribute for the interest in using those cells for personalised tissue regeneration.

In a previous study, we demonstrated the possibility to successfully gain human ADSC (hADSC) not only from subcutaneous fat tissue, but also from human visceral and omental fat tissue. [2]

For further clinical use of the cells it was relevant to know whether all the isolated cell types are able to form micromasses, three-dimensional cell cultures. Without an anchoring material like the bottom of a petri dish, cells use to congregate in order to form a so-called sphere, which displays both an in vitro tissue-model and a circumscribed three-dimensional source of tissue regeneration. Previous studies showed that cells being part of micromasses have an upregulated cell activity. Especially in micromasses of hADSC, a significant increase of angiogenic growth factors, as HGF, VEFG and FGF2, was found in comparison to a monolayer culture. [3] Furthermore, a strong influence on their differentiation capacity was observed, represented by the increase on the expression of specific markers for osteogenic (RUNX2), neurogenic (nestin), and hepatogenic differentiation (albumin). [4]

Placed on scaffolds like collagen membranes these well-organised spheres could be implanted into tissue defects.

There are different methods to develop micromasses: They can be centrifuged in order to obtain a single pellet, the hanging drop culture method can be applied to form many small micromasses, continuous rotating culture flasks like spinner flasks may form micromasses and culture surfaces can be coated with non-adhesive substances like agarose or chitosan films. [5–8]

Different cell types like osteoblasts, endothelial cells, or fibroblasts, but also ADSC were applied to form micromasses. [3, 4, 9–11]

The aim of the present study was to reveal the micromass-formation-potential of different hADSC types (subcutaneous, visceral and omental fat tissue derived cells) and to compare their histological attributes. Secondly we wanted to examine collagen membranes serving as scaffold for potential in-vivo application and related differences of subcutaneous, visceral and omental fat tissue derived micromasses in cultivation.

## Materials and methods

### Cell isolation and cultivation of hADSC

Human omental, visceral and subcutaneous fat tissue declared as waste product was obtained under sterile conditions by the General and Visceral Surgery, University Hospital, Muenster (Germany). This procedure had been approved by the ethical approval board of the University of Muenster, Germany. Cells were isolated as described in our previous study. [2] Each type of hADSC was obtained from three different and independent donors. Technical replicates were used in order to fortify results.

### Micromass-cultures

Micromasses of 200,000 cells were used for morphological evaluation. Therefore, cells suspended in α-MEM (Lonza Walkersville; USA) were plated into agarose coated 96-Well plates for 7 days. Micromasses were cultivated at 37 °C with 5% CO_2_; medium was changed every 2–3 days. Analysis was performed with three biological replicates.

### Cultivation of hADSC micromasses on collagen

hADSC micromasses were cultivated as described above. Collagen membranes (Resorba Wundversorgung GmbH & Co. KG, Germany) were cut to size of 0.8 cm × 0.8 cm and put into 8-well chamber slides (Nunc, Thermo Fisher Scientific, USA), filled with α-MEM. Single spheres were seeded on the soaked collagen. Micromasses were cultivated at 37 °C with 5% CO_2_, medium was replaced every 2–3 days.

### Histological examination of hADSC-micromasses

Micromasses were used for histological examination both exclusively and cultivated on collagen. Samples were fixed in 4% of buffered formalin (Fisher Scientific UK limited, UK) for 1 h and embedded in HistoGel (Thermo Scientific, Germany). Samples were watered for 1 h and dehydrated in an increasing alcohol series followed by incubation in warm cedar wood oil (Merck KgaA, Germany), warm paraffin - cedar wood mixture (ratio 1:1), and warm paraffin (Paraplast Plus) (Kendall, Tyco Healthcare Group LP, USA). After cooling down, samples were embedded into fresh paraffin for being sectioned with a microtome (Leica Microsystems GmbH, Germany). Sections were mounted onto slides one day before staining, and afterwards deparaffinized in xylene and rehydrated through decreasing grades ethanol solution. Primary monoclonal antibodies from mouse were CD13 (clone WM 15, dilution 1:100, Thermo Fisher Scientific, USA), CD44 (clone A3D8, dilution 1:100; Sigma Aldrich, Germany), and CD90 (clone AF-9; dilution 1:50; Thermo Fisher Scientific, USA). Dako REAL™ Detection Kit was used for secondary antibody detection (Dako, Germany). Haematoxylin was used for counterstaining (Sigma-Aldrich, Germany). Negative as well as positive controls were implemented according to manufacturer’s protocols. They were then examined utilising fluorescence microscope Axioplan 2 (Carl Zeiss, Germany). Staining results were summarised in a semi quantitative score defined as: 0 = no staining, 1 = staining in less than 30% of cells, 2 = staining in 31 to 80% of cells, 3 = staining in more than 80% of cells. Samples were analysed by three well-trained professionals with experience in histochemical techniques and analysis. Statistical analysis of semi quantitative score was carried out by one way ANOVA using a modified Levene testing and *p* < 0.05, and a PostHoc analysis with Bonferroni-Holm testing (Daniel’s XL Toolbox version 6.53; https://www.xltoolbox.net/. sourceforge.net).

### Cell tracking

In order to analyse micromass formation, amounts of 5000 hADSC suspended in Leibovitz’s L-15 medium (Gibco/ Life Technologies, USA) were plated into agarose (Biozym Scientific GmbH, Germany) coated 8-well plates. Using the ibidi-Heating-System (ibidi GmbH, Germany), the microscope camera DS-Fi1 (Nikon, Japan) and the software “micro trac” (PD. Dr. D. Dirksen, University of Muenster), the movement of cells was displayed with 2.5 times magnification.

## Results

**Fig. 1:**
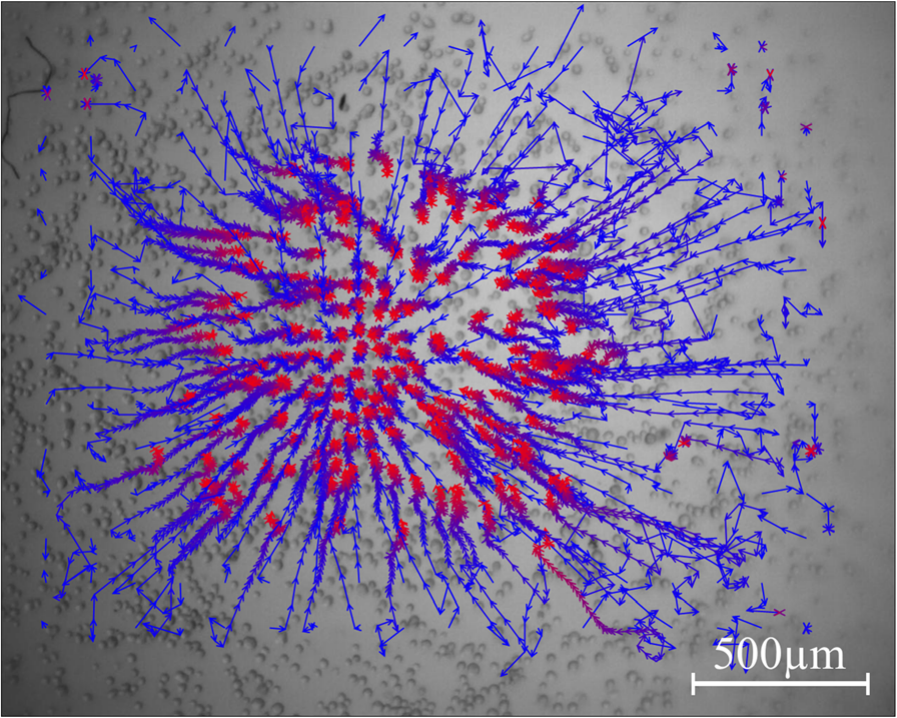
Cell tracking of subcutaneous fat tissue derived cells during micromass formation using the software Micro Trac (software: PD Dirksen, Uni Muenster; blue arrows = distance covered; red arrows = current movement)

**Fig. 2:**
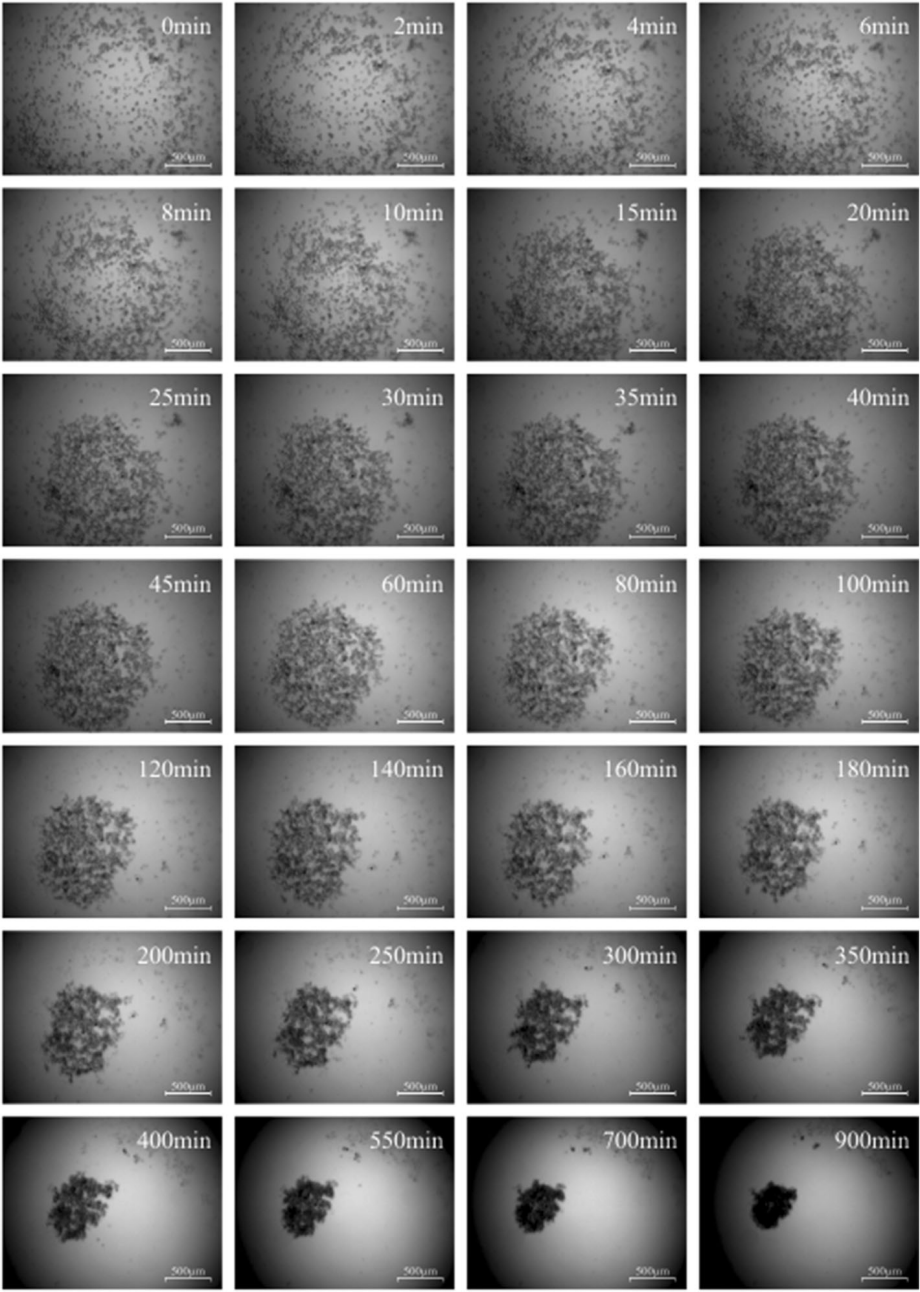
Micromass formation process of subcutaneous fat tissue derived cells (scale bar = 500 μm)

**Fig. 3:**
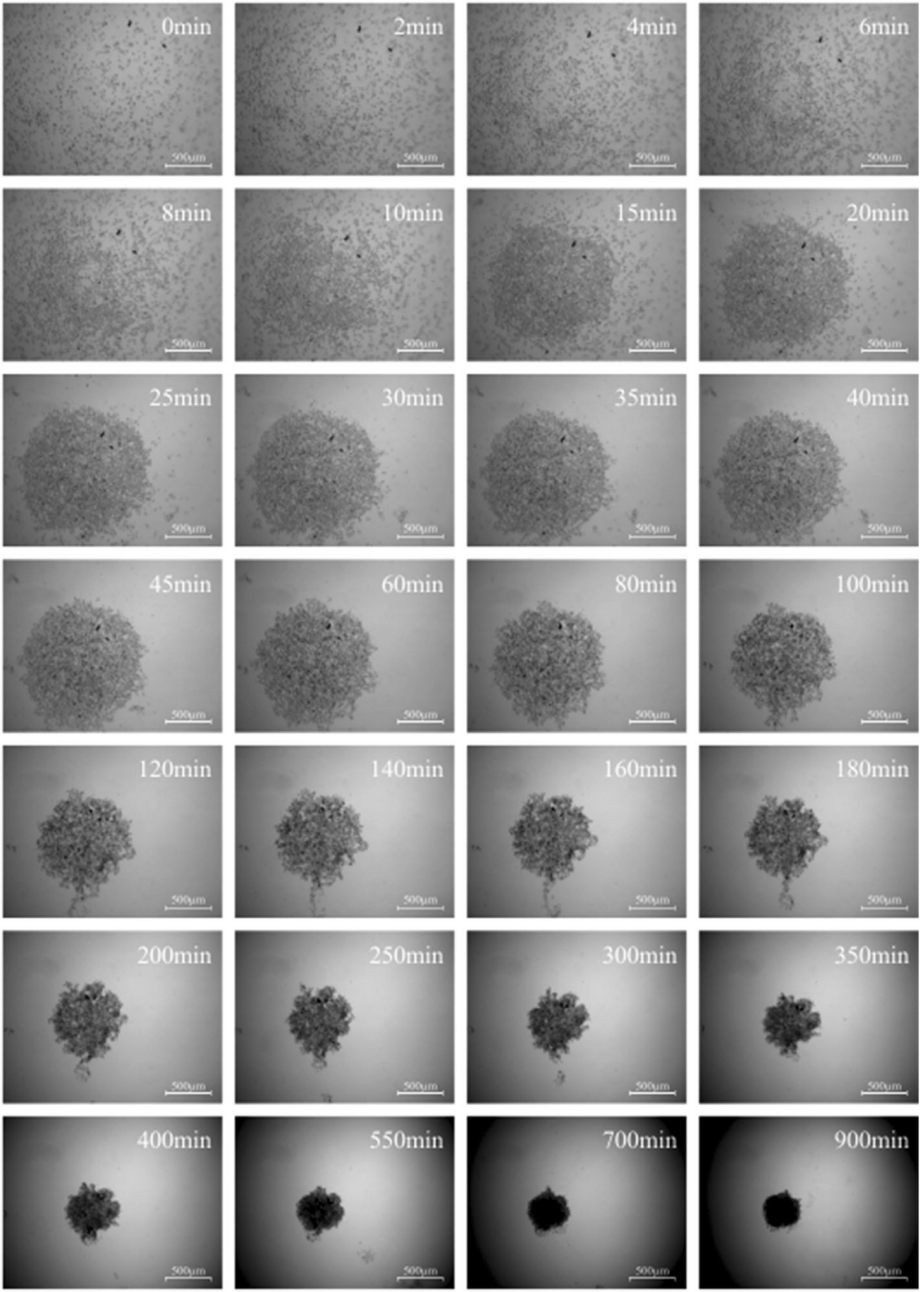
Micromass formation process of visceral fat tissue derived cells (scale bar = 500 μm)

**Fig. 4:**
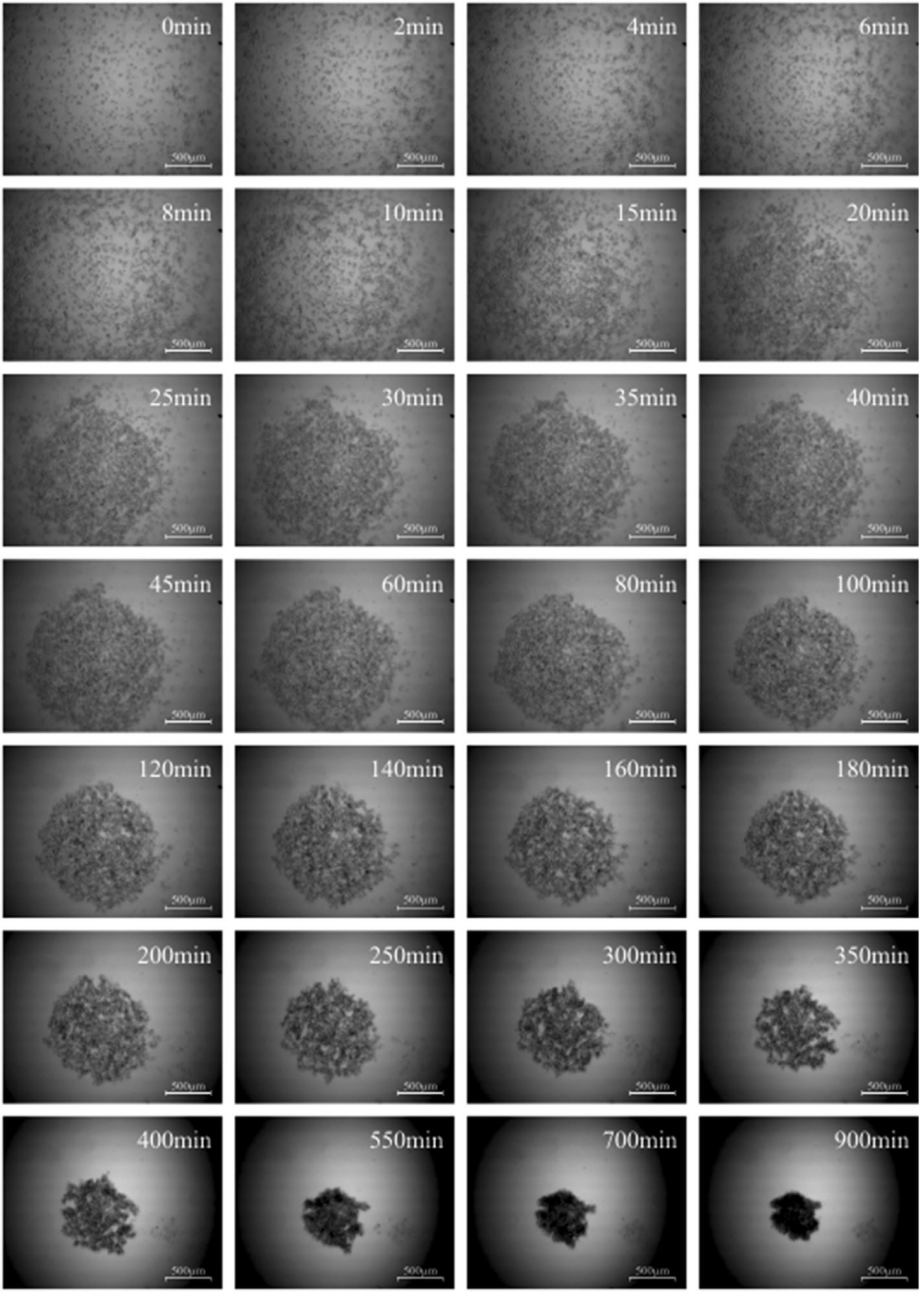
Micromass formation process of omental fat tissue derived cells (scale bar = 500 μm)

**Fig. 5:**
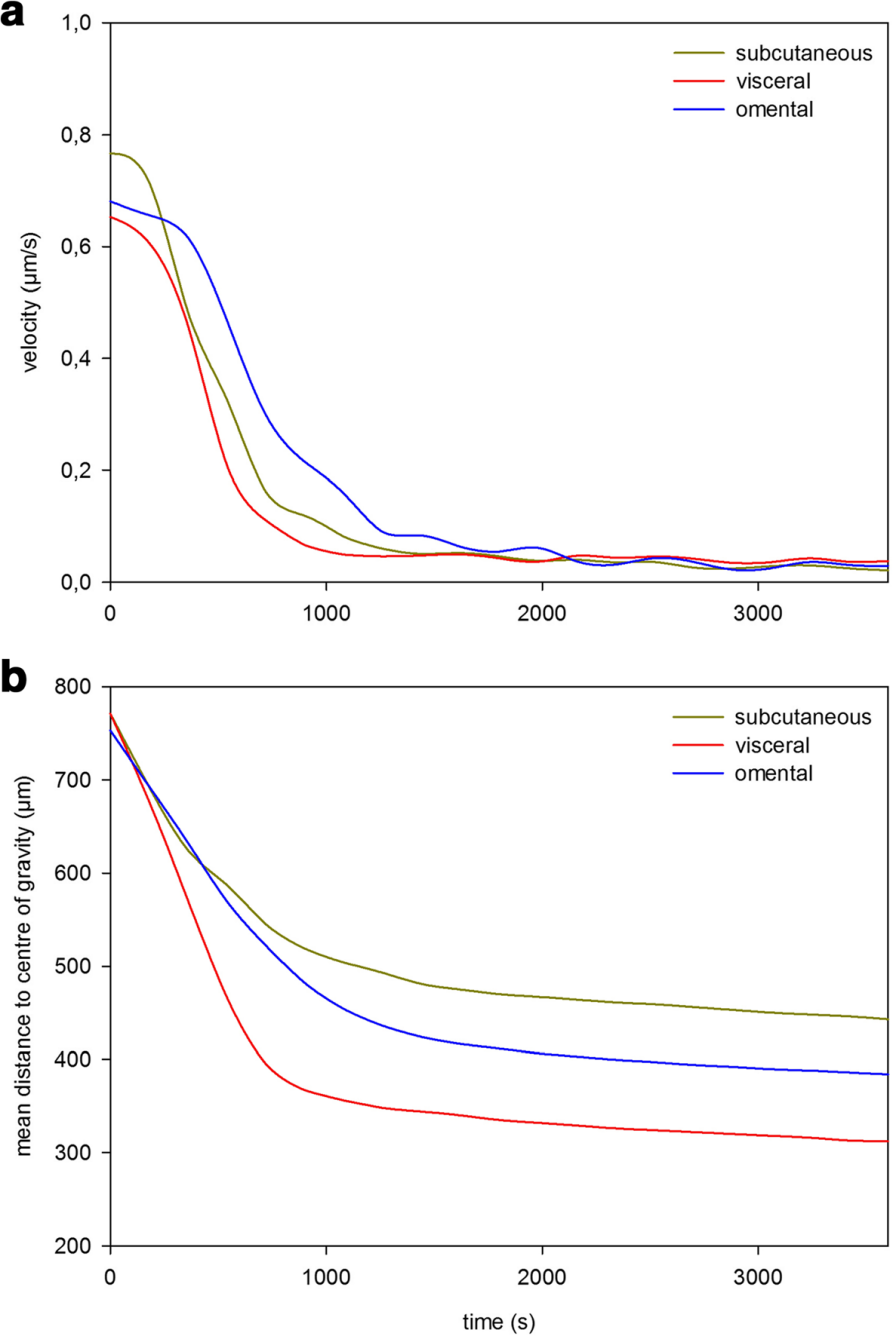
Mean velocity a) and distance to centre of gravity b) of subcutaneous, visceral and omental fat tissue derived cells during micromass formation process

Micromass formation of subcutaneous, visceral and omental fat tissue derived cells was displayed by the software “MicroTrac”. Every 5 s a picture was taken to work out the individual cell movement (Fig. 1). Subcutaneous fat tissue derived cells started to congregate after 3–5 min in order to form a spherical shape. The centre of gravity was located on every point of the well (Fig. 2). After 45 min of condensation the mean diameter of all cultures measured was nearly 1390 μm. The condensation process continued until a compact micromass culture with a mean diameter of nearly 471 μm had been formed (after 15 h). Visceral (Fig. 3) and omental (Fig. 4) fat tissue derived cells showed similar properties in comparison to subcutaneous ones, which was also revealed by cell tracking analysis. Within 45 min the majority of cell movement took place as cells formed a micromass culture with diameter averages of 1350 μm for visceral and 1476 μm for omental tissue. After 15 h, the condensed spheres diameter measured 400 μm for visceral and 493 μm for omental cells. The cell velocity and the cell’s distance to the centre of gravity decreased gradually. The cell movement compared to timeline is displayed in Fig. 5: the mean velocity of micromass formation (Fig. 5a) as well as the mean distance to center of gravity are shown (Fig. 5b). Most of the cell movement took place within the first 45 min, while all cell types showed nearly equal properties: cell velocity, which was very high initially, decreased continuously (Fig. 5a). The cells distance to the growing micromass centre (centre of gravity) is shown in Fig. 5b. Cells of all types of fat tissue congregate over the shortest possible path in order to form micromasses.

### Morphological characterisation of micromasses derived from subcutaneous, visceral and omental fat tissue cells

**Fig. 6:**
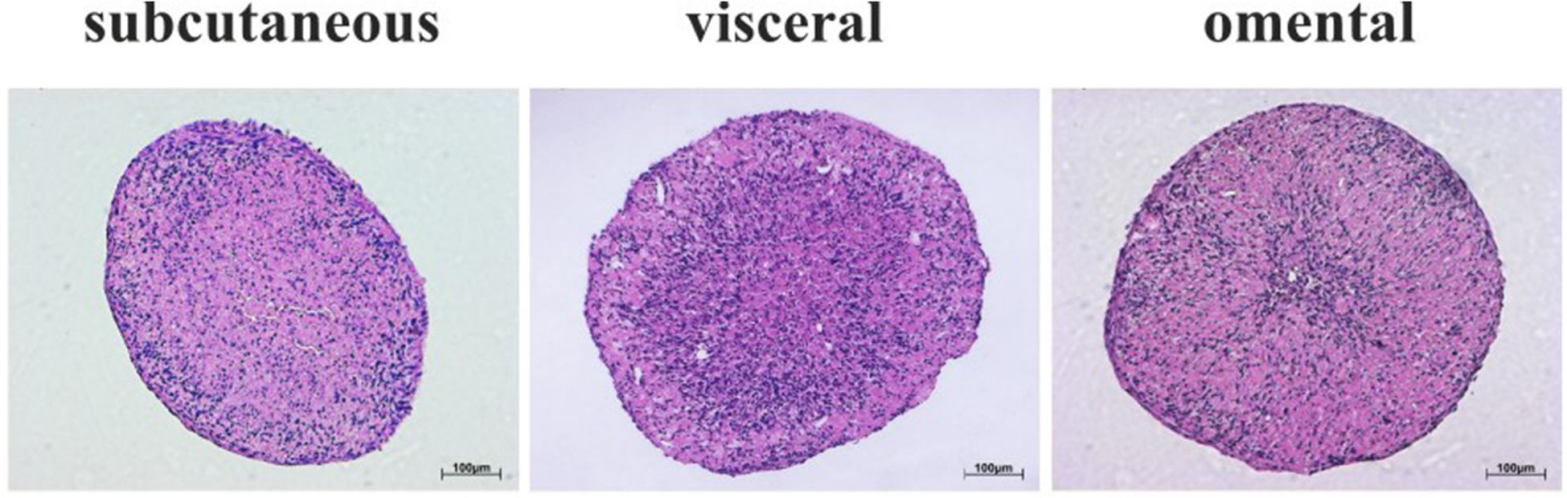
HE Staining of subcutaneous, visceral and omental fat tissue derived micromasses after 1 week of culturing. Subcutaneous micromasses are more homogeneous and compact as compared to visceral and omental ones

HE-Staining showed a homogeneous cell quality in subcutaneous fat tissue derived cells (Fig. 6). In the peripheral area the cells were compressed resulting in a higher density and reducted size whem compared to the centre. Deviating from this, micromasses consisting of visceral and omental cells fat tissue derived cells showed other included cell types and air locks as well. Both types were not as compact and homogeneous as spheres consisting of subcutaneous fat tissue derived cells. All the micromasses cultivated for one week showed an oval or round form. Mean diameter of round spheres were 1000 μm and for oval spheres 800 to 1200 μm.

### Cultivation of hADSC-micromasses on collagen membranes

**Fig. 7:**
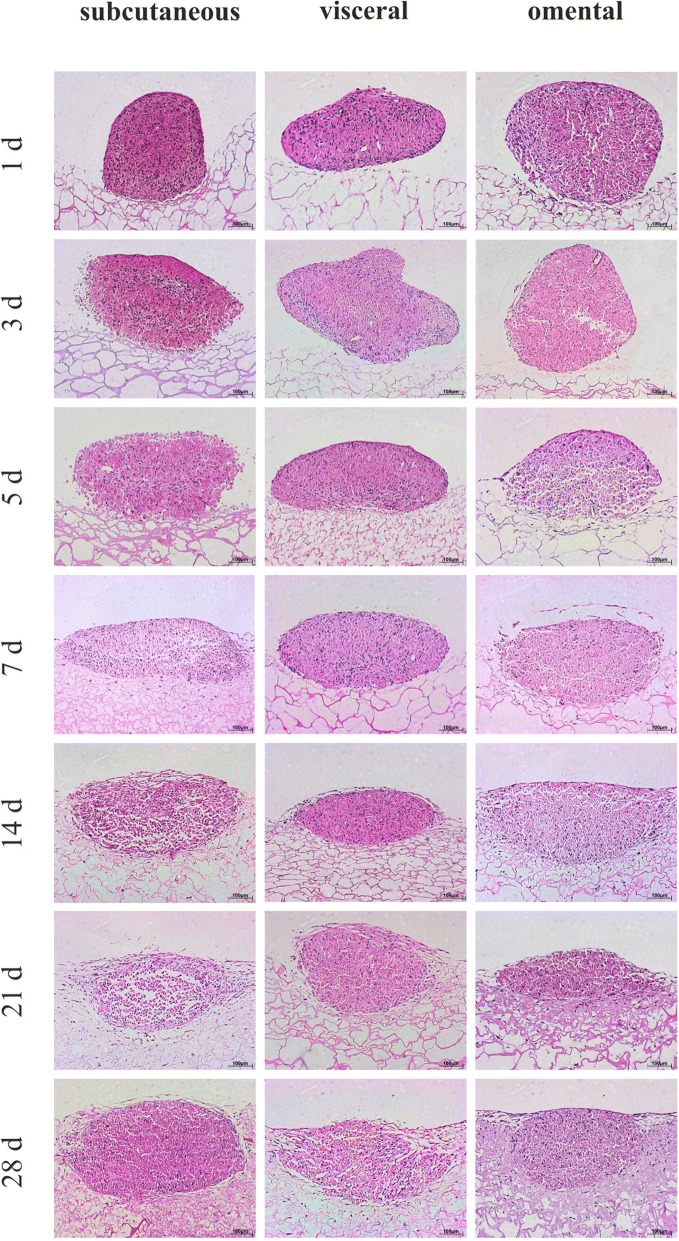
HE-Staining of cultivated subcutaneous, visceral and omental fat tissue derived cell-micromasses on collagen membranes

After seeding 7 day old spheres on collagen membranes, cells started to attach to the surface (Fig. 7). No differences in compound structure were observed between the three fat types hADSC. Within the following weeks the micromasses absorbed into the collagen membrane, while a spreading out of cells was visible after 5 days (Fig. 7). Spheres were totally absorbed into the collagen membrane after 14 to 28 days with the shapes of the spheres still being observable. Changes in expression of CD44, CD90, and CD13 of ADSC microspheres on collagen membrane were analysed with IHC staining and a semi quantitative score was defined. The results were summarised in Table 1. One way ANOVA was accomplished and differences were analysed on a level of significance of *p* < 0.05; referring to score of CD44 with a *p*-value of 0.0044, referring to score of CD90 with a p-value of 0.046, and referring to score of CD13 with a p-value of 2.9 × 10^− 6^. Also a PostHoc test was performed. There were significant differences for CD44 between omental and visceral cells (*p* = 0.0085) such as between omental and subcutaneous cells (*p* = 0.002). Furthermore, a statistical differences was found for CD13 between subcutaneous and visceral cells (*p* = 5.23 × 10^− 5^) and between subcutaneous and omental cells (*p* = 1.7 × 10^− 4^).

**Table 1:** Semi quantitative IHC score of stem cell markers in hADSC on collagen scaffolds (standard deviation in brackets)

	hADSC from subcutaneous fat		hADSC from visceral fat		hADSC from omental fat	
CD44						
Day 1	2.2	(±0.1)	3	(±0)	1.3	(±0.4)
Day 7	2.7	(±0.4)	2.5	(±0)	0.8	(±0.2)
Day 14	1.7	(±0.4)	2	(±0.2)	0.8	(±0.2)
Day 28	0	(±0)	1	(±0)	0	(±0)
CD90						
Day 1	1	(±0.6)	1.3	(±0.2)	3	(±0)
Day 7	2.2	(±0.6)	2.4	(±0.4)	2.3	(±0.3)
Day 14	1.3	(±0.4)	1.7	(±0.1)	2.5	(±0)
Day 28	0.7	(±0.4)	1.3	(±0.2)	1	(±0.4)
CD13						
Day 1	2.3	(±0.4)	0.8	(±0.2)	1	(±0)
Day 7	1. 7	(±0.4)	1.4	(±0.3)	1	(±0)
Day 14	2.3	(±0.4)	1	(±0.1)	1	(±0)
Day 28	2.3	(±0.4)	0.8	(±0.6)	0.8	(±0.2)

The expression of CD44 decreased gradually in hADSC micromasses of all three fat types. After 28 days, it was no longer expressed in hADSC derived from omental fat (Tab. 1). The expression of CD90 also decreased in time, but it was detectable after 28 days. Also the expression of CD90 increased during the first weeks in hADSC micromasses derived from subcutaneous and visceral fat (Tab. 1). The expression of CD13 remained constant with slight fluctuations (Tab. 1).

## Discussion

To date, the micromass technique has been an approved method to understand the basic organisation of cells within three-dimensional tissues, offering a prospective view towards scaffold-free applications in regenerative concepts. ADCS micromasses are usually known for their differentiation towards a chondrogenic lineage in order to mimic the structure of the cartilage.[11–14] However, any other monolayer differentiation can be applied as micromass culture as well, e.g. neurogenic, osteogenic or adipogenic differentiation. [15–17] A previously described, elaborated method for creating ADSC micromasses is the use of a non-adhesive primary layer technique. Compared to other cultivation techniques such as the “hanging drop culture method” or spinner flasks, it allows a specific control during the aggregation of cells and enables easy and inexpensive creation of voluminous micromasses for defined points in time. Polysaccharides like chitosan and agarose are mentioned as non-adhesion primary layer. In this study we used agarose to create micromasses - a technique that was first mentioned in 1984. [4, 5, 18–20]

As described previously, hADSC derived from subcutaneous, visceral and omental fat tissue share immunohistochemical characteristics but differ in morphological aspects. [2] In the current study, we showed that micromasses of three fat origins develop the same way. In 2013 Schäfer et al. observed an equal behaviour of 5000 bovine osteoblasts in forming micromasses. This may indicate that the chosen cell type has any or only little influence on the formation process of micromasses on agarose coated wells. [21]

In the current study, we showed that the morphology of subcutaneous, visceral and omental fat tissue derived micromasses differs in cell density and the inclusion of other cell types. Subcutaneous fat tissue derived spheres may appear homogeneous because of their abundance in the origin tissue, which can be easily harvested. As described in our previous study, visceral and omental fat tissue are both contaminated by fibroblasts or mesothelial cells which may influence the sphere’s morphology displayed in HE-staining. Dying mesothelial cells, which could not be cultivated on the used α-MEM cultivation media, may caused air locks. Nevertheless, all cells were able to create solid micromasses, surrounded by an outer layer of thin cells. In the study of Neunzehn et al. on which osteomicrospheres were observed, this border layer was also visible, suggesting an epithelial function. [22]

A further clinical advantage of micromasses was described by Liu et al. in 2013, who reported that ADSC spheroids, aggregated on a non-adhesive primary layer as chitosan, appeared to change cell characteristics. [19] ADSC micromasses, which are cultivated under hypoxic conditions, secrete a high amount of anti-apoptotic factors and lower levels of pro-apoptotic factors. [3] Laschke et al. described a higher level of angiogenesis in murine ADSC micromasses, created on agarose, which were implanted as a part of scaffolds in the dorsal skin of mice. [20]

Concerning various cell types, other studies revealed similar results of higher cell proliferation and differentiation in three-dimensional systems as compared to monolayer cultures, which are affected by a different cell-cell and cell-matrix interaction. [23–25] As described by Gerber et al. osteoblast-like cells cultured in a micromass system also show a higher level of cell differentiation and mineralisation. [26] Three-dimensional cultivated cells seem to have a higher resemblance to physiological organism as monolayer cultivating methods. [27]

In the current study, hADSC micromasses were plated onto membranes of collagen, which is a naturally produced protein in the organism. Previous studies showed various types of stem cells seeded onto collagen membranes as a prospective approach in tissue engineering. [28–30] We showed that there are no differences in miscellaneous fat tissues. As a result, we consider subcutaneous as well as visceral and omental derived cells a promising material for the treatment of tissue defects. The observed spreading of cells into the surrounding tissue highlights the high biological activity of this scaffold-cell-complex.

The proximity of cells in micromasses affects cell-cell interaction, resulting in an increase of expression of specific reporter molecules. [31] The expression of specific angiogenic and antiapoptotic factors in hADSC spheres under inducing cultivation medium was at a 20-times higher level as in monolayer cultures. [3]

In this study, changes on the expression of characteristic surface markers in hADSC were analysed. [2] The following markers were analysed, since these are the most prominent markers for hADSC: CD13, expressed especially in endothelial cells of the intestines, CD44 and CD90, an essential glycoprotein with substantial importance in cell-cell adhesion. [32–35] Cheng et al. observed a higher expression of CD44 and a lower expression of the mesenchymal marker CD90 in subcutaneous fat tissue derived micromasses. [4] They suppose that ADSC cultured as micromasses shift away from their mesenchymal line in order to reach a more primitive state. [4] Our study showed an obverse expression of CD44 and CD90. The expression rate of CD44 decreased during time and an outgrowing into collagen matrix and was not detectable after 28 days. The expression of CD90 increased during the first weeks. An increasing rate of CD90 in ADSC micromasses was also observed in other studies. [36]

Regarding to the formation of an environment similar to the physiological one, the micromass technique appears to be not only an insightful visualisation of cellular processes within living tissue, but also an auspicious prospect in the therapy of tissue defects. Hence, due to the differentiation capacity of hADSC their use seems to be particularly promising to generate damage tissues. Our findings revealed morphological differences in hADSC micromasses with subcutaneous fat tissue derived cells showing optimal results, but displayed similarities in formation process and ability to adhere to a biological surface like collagen. Although further investigations in animal models are required, these similarities may support existing theories of ADSC based regenerative concepts, which could potentially be applied in autologous tissue regenerating.

## Conclusion

### Clinical significance

Surgical procedures are often accompanied by hard and soft tissue-defects. For its repair, we presented three types of fat tissue as a multipotent stem cell reservoir. Micromasses in combination with a scaffold like collagen presents a solid, robust, and good manageable cell conglomerate to fill-out tissue defects.

### Micromass formation and morphology

Micromass formation of all three analysed fat tissues could be achieved. Formation process was similar between fat types and observed differences were slightly.

### Cultivation on collagen

Micromasses of all three fat types adhered to collagen and were incorporated into collagen matrix. Expression of stem cell markers common used in hADSC changed during cultivation. These are first hints for a beginning de-differentiation of hADSC.

In conclusion, hADSC derived from subcutaneous fat tissue appearing to be the best material for regeneration concepts.

## Abbreviations

ADSC: Adipose derived stroma cells
h: Human
HE: haematoxylin and eosin
IHC: Immune histochemical
